# Laser Direct Metal Deposition of 2024 Al Alloy: Trace Geometry Prediction via Machine Learning

**DOI:** 10.3390/ma11030444

**Published:** 2018-03-19

**Authors:** Fabrizia Caiazzo, Alessandra Caggiano

**Affiliations:** 1 Department of Industrial Engineering, University of Salerno, 84084 Fisciano (SA), Italy; f.caiazzo@unisa.it; 2 Department of Industrial Engineering, University of Naples Federico II, 80125 Naples, Italy; 3 Fraunhofer Joint Laboratory of Excellence on Advanced Production Technology (Fh-J_LEAPT UniNaples), 80125 Naples, Italy

**Keywords:** laser direct metal deposition, aluminum alloy, machine learning, artificial neural network

## Abstract

Laser direct metal deposition is an advanced additive manufacturing technology suitably applicable in maintenance, repair, and overhaul of high-cost products, allowing for minimal distortion of the workpiece, reduced heat affected zones, and superior surface quality. Special interest is growing for the repair and coating of 2024 aluminum alloy parts, extensively utilized for a wide range of applications in the automotive, military, and aerospace sectors due to its excellent plasticity, corrosion resistance, electric conductivity, and strength-to-weight ratio. A critical issue in the laser direct metal deposition process is related to the geometrical parameters of the cross-section of the deposited metal trace that should be controlled to meet the part specifications. In this research, a machine learning approach based on artificial neural networks is developed to find the correlation between the laser metal deposition process parameters and the output geometrical parameters of the deposited metal trace produced by laser direct metal deposition on 5-mm-thick 2024 aluminum alloy plates. The results show that the neural network-based machine learning paradigm is able to accurately estimate the appropriate process parameters required to obtain a specified geometry for the deposited metal trace.

## 1. Introduction

In recent years, additive manufacturing processes, characterized by layer upon layer construction of parts, have emerged as an alternative to conventional processes for the manufacturing of various metal materials like steel, Inconel, aluminum and titanium alloys [1,2,3,4].

Due to progress in computation power and systems technology, laser-based additive manufacturing, employing a laser beam to provide thermal energy for the melting and consolidating of the additive materials, has notably advanced and is receiving a great deal of attention due to its high potential for industrial applications [1,2]. The most common laser-based additive manufacturing processes use powder material include selective laser sintering, laser beam melting, and laser direct metal deposition. Selective laser melting (SLM) has been employed for processing various metal alloys for the biomedical, aerospace and automotive industries, being able to produce complex-shape parts in a highly efficient way and to provide properties comparable or superior to those produced by other conventional methods [3,5]. 

Laser direct metal deposition (DMD) is an advanced additive manufacturing technology which is attracting increasing interest due to its suitable applicability in maintenance, repair and overhaul of critical high-cost products, such as those employed in the aerospace and automotive industry. These complex products may be subject to manufacturing-induced damages or to severe operating conditions (temperature, wear, and mechanical stresses) hindering the product’s operational functionality: in both cases, to avoid scrap part replacement, which create high costs, proper part recovery operations are required.

In laser direct metal deposition, a laser beam is used as a focused heat source to scan the surface and create a melting pool over an existing metal substrate. Since the added metal impinging the molten pool is fed simultaneously with the laser action (i.e., in single stage processing) in the form of wire or loose powder, a deposited metal trace is generated with metallurgical bonding to the substrate as a result of fusion and diffusion phenomena [6]. 

Single weld tracks are placed next to each other in order to form a single layer with thickness varying from 0.1 mm to several millimeters depending on the process parameters (velocity, powder feed rate, and laser power) [1,7]. In order to coat wide surfaces on 3D complex geometries, side overlapping of the individual laser traces is required. The process can be utilized in the repair of worn out high value components, the building of new components, and the application of wear resistant and corrosion resistant coatings [8].

In comparison with conventional coating and repair techniques such as arc welding and plasma spraying, which involve a large temperature increase in the part and a weakening of the base as a result of the wide temperature distribution in the working region, laser direct metal deposition allows for minimal distortion of the workpiece, reduced heat affected zones (HAZs), and superior surface quality [1,9]. Moreover, the coating adherence and its tribological behavior are reported to be higher in laser DMD [10]. Another interesting aspect of DMD technology is the possibility to achieve enhanced productivity, higher process automation, and reduction of the overall processing time, which are main targets within adaptive and flexible manufacturing environments typical of the factories of the future [11].

At present, two possible DMD feedstock kinds are offered: wire and powder. The former is generally preferred for its lower cost and inferior probability of oxide content [12], whereas the latter is preferred for its flexibility in material selection and the higher precision for local repair [13] with better surface quality and bonding strength [6,14]. 

As regards the substrate materials to be processed via laser direct metal deposition, interest is growing for the repair [15] and coating [16] of 2024 aluminum alloy components. This alloy is characterized by excellent plasticity, corrosion resistance, electric conductivity, and strength-to-weight ratio. For this reason, among all of the commercial high strength heat-treatable and age-hardenable aluminum alloys, the 2024 alloy is the most extensively utilized for a wide range of applications and manufacturing areas, especially in the automotive, military, and aerospace industries.

In this research work, laser direct metal deposition of 2024 Al alloy is investigated through an experimental campaign under different process conditions carried out on 5-mm-thick T3 temper 2024 Al alloy plates, generating single deposited metal traces with a length of 100 mm.

On the final part, many quality parameters can be of interest, including material density, mechanical material properties, surface quality, and dimensional and geometrical accuracy [1]. With reference to dimensional and geometrical accuracy, the objective of this research work is the prediction of the resulting geometry of the deposited metal trace cross-section, which is a critical issue related to the laser metal deposition process. In the literature, research efforts have been spent to predict the cross-section geometry of the metal deposition via mathematical modeling based on prior experimental work. El Cheikh et al. [17] implemented a mathematical model to predict the clad cross-section dimensions and obtain an analytical description of the clad geometry, establishing analytical relationships between the radius and the centre of the disk-shaped clad cross-sections and the process parameters [17]. Since a number of processing parameters are involved in laser direct metal deposition, statistical analysis of the responses was employed in other research works [18,19]. 

In the present work, an alternative approach to deal with the complexity of the laser direct metal deposition modelling was proposed. A machine learning paradigm based on artificial neural networks (ANN) was developed to find correlations between the input laser metal deposition process parameters and the output geometrical parameters of the deposited metal trace produced by laser DMD of 2024 aluminum alloy. Machine learning is an artificial intelligence method which allows for data-driven formulation of complex models that lend themselves to predictions or decisions from sample inputs revealing the structural patterns embedded in data [20,21]. In this study, the specific aim of the machine learning approach is to estimate via ANN the appropriate process parameters required to obtain a deposited trace with a cross-section of given geometrical parameters. The results of the experimental campaign of laser DMD of 2024 Al alloy are employed in order to train the ANN according to a two-phase procedure for the estimation of the appropriate process parameters and following verification.

## 2. Experimental Procedure

**Figure 1:**
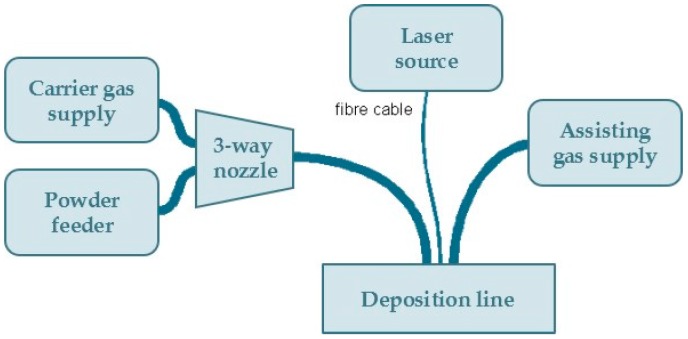
Main components in the laser deposition line.

The experimental campaign of laser direct metal deposition on 2024 Al alloy plates was carried out using a laser deposition line, which is a complex system comprising several basic components, as shown in Figure 1.

**Figure 2:**
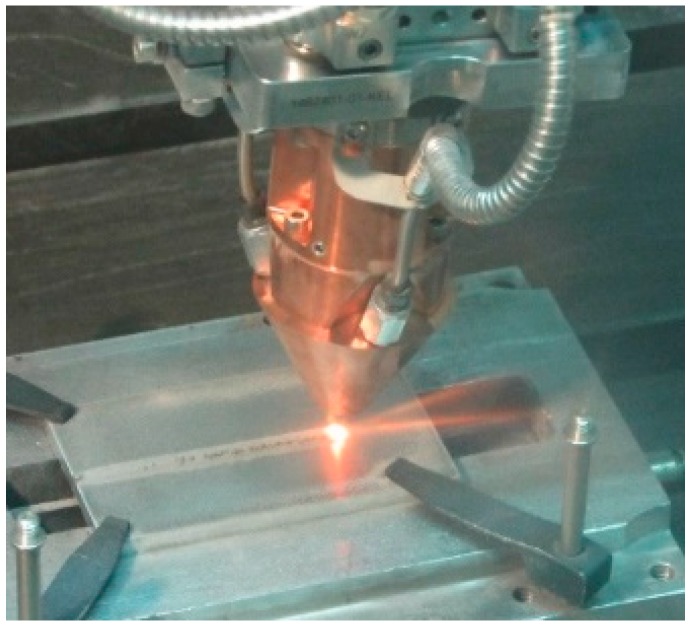
Laser head performing direct metal deposition.

A fiber-delivered Yb:YAG disc laser source operating in continuous wave emission with the technical features reported in Table 1 was employed. The displacement of the laser head is accomplished by a six-axis industrial robot with dedicated controller; an in-built feeding nozzle is displaced together with the laser head (Figure 2).

**Figure 3:**
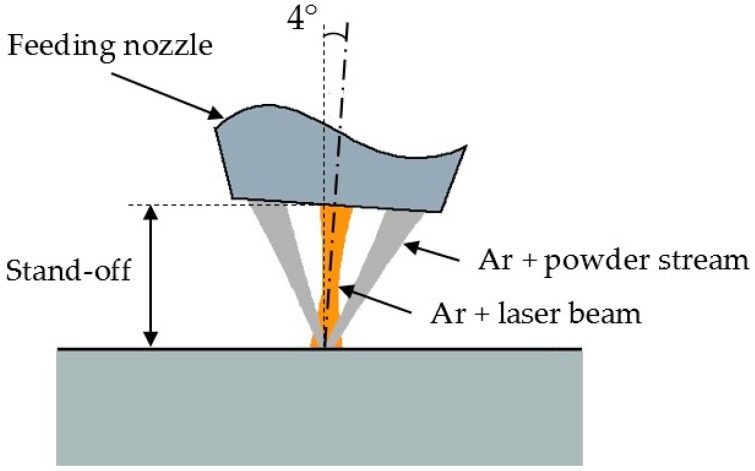
Scheme of the three-way feeding nozzle.

To supply the metal powder, a three-way feeding nozzle was used, which receives the metal powder from a feeder with an oscillating conveyor (Figure 3). Namely, three stream cones of metal powder enclosing the laser beam are provided; each stream is injected by its separate argon conveying flow. Argon gas, flowing coaxially with the laser beam, was employed to shield the melting pool from the environment. In agreement with the common practice in processing highly reflective metals such as aluminum and copper alloys, a tilting angle of 4° was used for the laser head to prevent back-reflections from entering the optical train. 

With the scope to repair real components using materials with the same or similar characteristics as the substrate metal, 2024 aluminum alloy powder was utilized in this study. The spherical shaped powder had particle sizes ranging between 20 and 60 µm, as certified by the powder supplier. Since a steady feeding rate must be provided constantly, the powder was preliminary dried in furnace at 180 °C for 2 h so as to properly flow via the conveyor. 

The workpieces to be processed consisted of 5-mm-thick 2024 aluminum alloy plates in T3 heat treatment state. The laser DMD process was performed so as to produce cladding traces in the form of single metal deposition along an overall scanning length of 100 mm under diverse process conditions.

Since numerous process variables are involved in direct metal deposition, a systematic approach was employed to select the process conditions of the experimental campaign. The main process parameters of the experimental plan were selected with reference to the literature and past experience: namely, laser power, *P*, scanning speed, *v*, and powder feeding rate, *ṁ*, were considered.

The thermophysical properties of the material, such as thermal conductivity, thermal expansion coefficient and melting point, have a significant influence on the geometry and mechanical characteristics of the deposition. However, since in this research work only one type of material was employed, both for the substrate as well as the powder, these properties remained constant in the experimental campaign and therefore they were not considered in the implementation of the ANN methodology.

Laser power was varied among five different levels comprised between 1200 W and 3000 W, scanning speed was varied among seven different levels between 150 and 600 mm/min, and powder feeding rate was varied among seven different levels between 4 and 10 g/min. For each process condition, three repetitions were carried out, and they were performed in random order so as to reduce systematic errors. 

**Figure 4:**
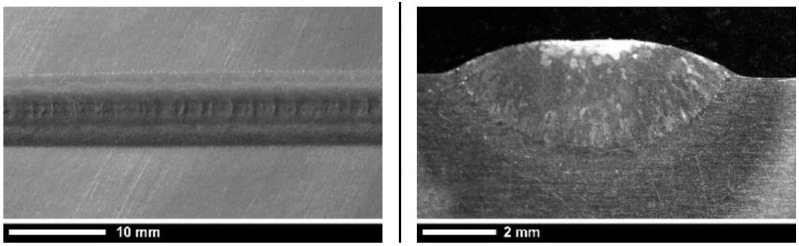
Deposited metal trace view and cross-section (*P* = 3 kW, *v* = 200 mm/min, *ṁ* = 4 g/min).

In order to evaluate the geometry of the deposited metal trace cross-section, cross-cutting and mechanical preparation was carried out on three samples for each process condition. Polishing to mirror finish and chemical etching with a solution of 10% hydrofluoric acid and 15% nitric acid in water at room temperature were performed. Figure 4 shows the view and the cross-section of a deposited metal trace produced by one of the experimental tests of laser DMD carried out at *P* = 3 kW, *v* = 200 mm/min, *ṁ* = 4 g/min. 

**Figure 5:**
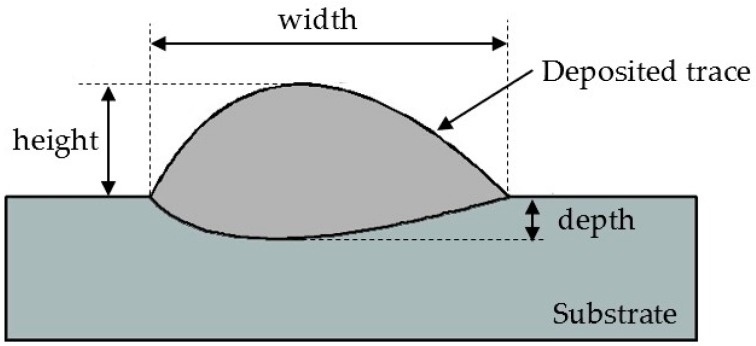
Scheme of the deposited trace cross-section geometrical parameters.

The characteristic geometrical parameters of the deposited trace cross-section were measured as indicated in Figure 5. The measurement values for each process condition are reported in (Table 2).

## 3. Artificial Neural Network-Based Machine Learning for Process Parameters Estimation

In this research work, a machine learning paradigm based on artificial neural networks (ANN) was developed to find correlations between the input laser direct metal deposition process parameters (laser power, *P*, scanning speed, *v*, and powder feeding rate, *ṁ*) and the output geometrical parameters (width, *w*, depth, *d*, and height, *h*) of the deposited trace produced by direct metal deposition (Figure 5). 

The specific aim was to estimate via ANN the appropriate process parameters required to obtain a deposited trace with given *w*, *d*, and *h* geometrical parameters. The results obtained through the experimental campaign were employed to train the ANN according to a two-phase procedure, the first for the estimation of the appropriate process parameters, and the second for verification purpose.

### 3.1. ANN Data Processing for Process Parameters Estimation

In the first phase, the geometrical parameters (width, depth, and height) of the deposited metal trace produced by each experimental test were combined to construct feature pattern vectors (FPV) to be fed as input for ANN learning [22]. Due to the fact that 30 different combinations of laser DMD process parameters were experimentally tested, and each experimental condition was repeated three times, a training set of *n* = 90 FPV (one FPV for each experimental test) was generated.

FPV*_i_* = [*w_i_*, *d_i_*, *h_i_*]    *i* = 1, …, *n* (1)

To perform supervised ANN learning, each three-features FPV_i_ was associated to the corresponding process parameters combination (*P_i_*, *v_i_*, *ṁ_i_*), representing the output of the ANN.

Output*_i_* = [*P_i_*, *v_i_*, *ṁ_i_*]    *i* = 1, …, *n* (2)

As regards the ANN architecture, a three-layer cascade-forward backpropagation ANN was set up with a number of input layer nodes equal to 3, i.e., matching the number of input features, and a number of output layer nodes equal to 3, i.e., corresponding to the number of output process parameters. Different numbers of hidden layer nodes were tested, from 1× to 3× the number of input layer nodes, with the aim to identify the optimal ANN architecture providing the best performance rate. The Levenberg–Marquardt algorithm was selected as ANN training function.

In ANN learning, the training subset is utilized for calculating the gradient and updating the ANN weights and biases, while the testing subset is used to evaluate the performance of the trained ANN [22,23]. 

In this research work, the training set of *n* = 90 FPV was employed to train the ANN, while the testing procedure was performed using a different FPV set constructed using the average values of the geometrical parameters (width, depth, and height) of the deposit calculated over the three repetitions of each experimental condition. Since 30 different experimental conditions were tested, the testing set, built with the average values of measured deposit width, depth, and height, was made of *p* = 30 FPV. The overall pattern recognition performance over the testing set was eventually estimated by aggregating the *p* recognition rates obtained. 

The pattern recognition performance of each ANN architecture was evaluated in terms of root mean square error (RMSE) between ANN predicted values (*ŷ_t_*) and target values (*y*).

(3) $$RMSE\;=\;\sqrt{\frac{\sum_{t=1}^{n}{\left({\hat{y}}_{t}-y\right)}^{2}}{n}}$$

The best performance was obtained for the ANN architecture with a number of hidden layer nodes equal to nine, i.e., three times the number of hidden layer nodes. The aggregated RMSE values provided by this architecture over the *p* = 30 testing cases were equal to 61.6 W for power, 18.0 mm/min for speed, and 0.38 g/min for powder feeding rate.

**Figure 6:**
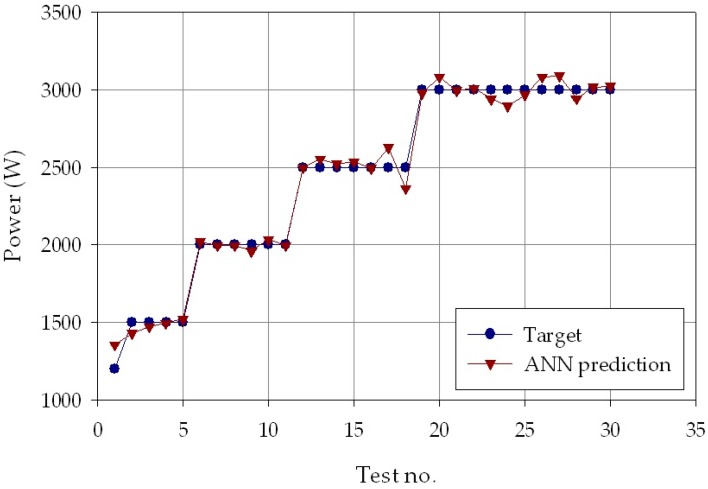
Experimental (blue marks) and ANN estimated (orange marks) power values, *P*, for each of the *p* = 30 experimental conditions.

**Figure 7:**
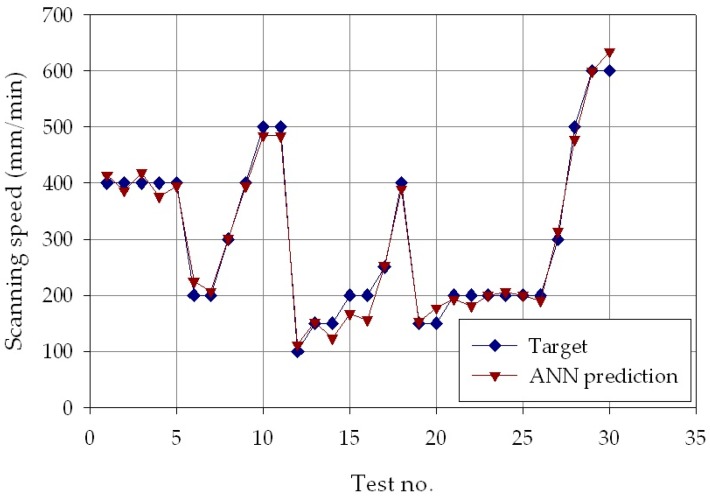
Experimental (blue marks) and ANN estimated (orange marks) speed values, *v*, for each of the *p* = 30 experimental conditions.

**Figure 8:**
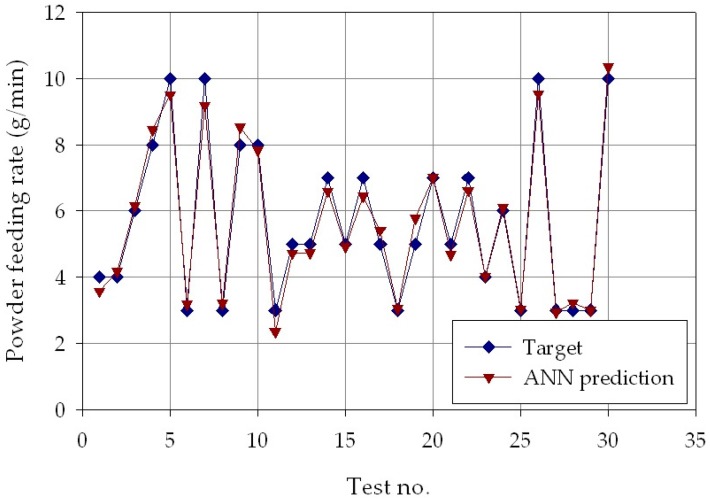
Experimental (blue marks) and ANN estimated (orange marks) powder feeding rate, *ṁ*, for each of the *p* = 30 experimental conditions.

The results are shown in Figure 6, Figure 7 and Figure 8, which report, for each of the *p* = 30 experimental conditions, the experimental laser direct metal deposition process parameters (laser power, *P*, scanning speed, *v*, and powder feeding rate, *ṁ*) and the process parameters estimated by the ANN with nine hidden layer nodes. From the Figures, it can be clearly observed that the experimental and the estimated values are very close to each other, indicating that the neural network evaluation was accurately performed. The mean absolute percentage error of the ANN estimation was as low as 2.0% for laser power, 5.8% for scanning speed, and 5.5 % for powder feeding rate.

### 3.2. ANN Data Processing for Geometrical Parameters Verification

The second phase of the ANN procedure was employed to verify the results provided by the ANN in the first phase, by performing the reverse data processing, i.e., estimating the geometrical parameters (width, depth, and height) of the deposit produced using the process parameters estimated in the first ANN phase.

In this phase, the laser DMD process parameters (laser power, *P*, scanning speed, *v*, and powder feeding rate, *ṁ*) were combined to build up the feature pattern vectors (FPV) to be fed as input for ANN learning.

As 30 different combinations of laser direct metal deposition process parameters were experimentally tested, and each experimental condition was repeated three times, also in this case the training set was made of *n* = 90 FPV, one FPV for each experimental test.

FPV*_i_* = [*P_i_*, *v_i_*, *ṁ_i_*]    *i* = 1, …, *n* (4)

To perform supervised ANN learning, each three-features FPV_i_ was associated with the corresponding deposit geometrical parameters, representing in this case the output of the ANN.

Output*_i_* = [*w_i_*, *d_i_*, *h_i_*]    *i* = 1, …, *n* (5)

Three-layer cascade-forward backpropagation ANN were setup with the same architectures and transfer function utilized for the first phase. 

As regards the testing FPV set, it was constructed using as input the *p* = 30 combinations of process parameters (*P*, *v*, *ṁ*) estimated by the ANN in the first phase. The ANN output during the testing phase should provide estimated values of the deposit geometrical parameters (width, depth, and height) sufficiently close to the average values provided as input in the first phase. The overall pattern recognition performance over the testing set was eventually estimated by aggregating the *p* recognition rates obtained. 

As before, the pattern recognition performance of each ANN architecture was evaluated in terms of root mean square error (RMSE) between ANN predicted values (*ŷ_t_*) and target values (*y*).

The best performance was obtained for the ANN architecture with a number of hidden layer nodes equal to nine, i.e., three times the number of hidden layer nodes. The RMSE values provided by this architecture over the *p* = 30 testing cases were equal to 0.59 mm for the deposit width, 0.53 mm for the deposit depth, and 0.14 mm for the deposit height.

**Figure 9:**
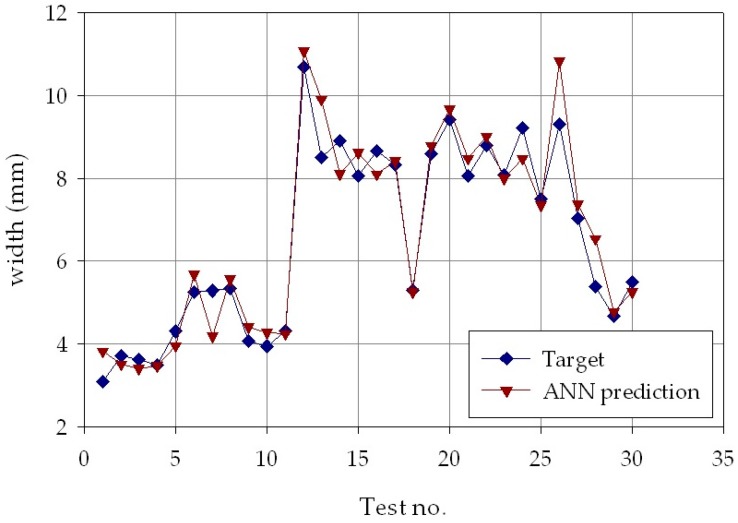
Experimental average (blue marks) and ANN estimated (orange marks) deposit width, *w*, for each of the *p* = 30 experimental conditions.

**Figure 10:**
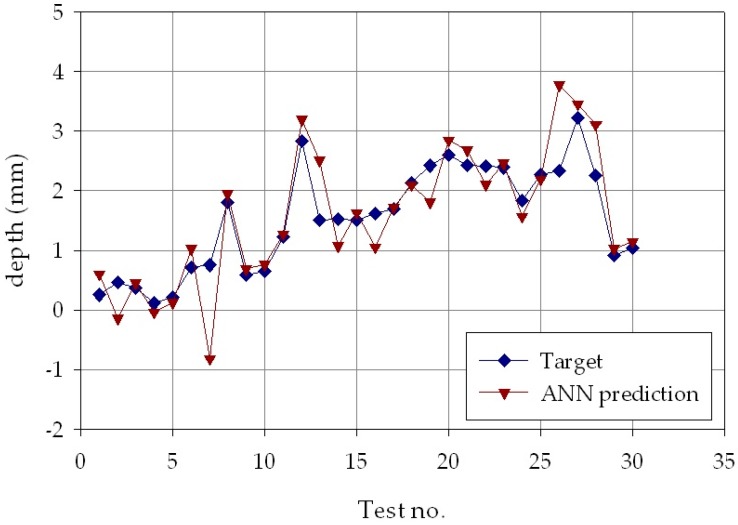
Experimental average (blue marks) and ANN estimated (orange marks) deposit depth, *d*, for each of the *p* = 30 experimental conditions.

**Figure 11:**
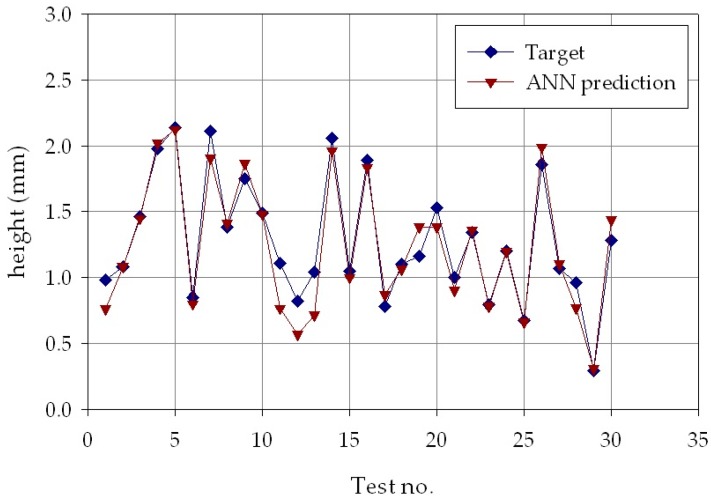
Experimental average (blue marks) and ANN estimated (orange marks) deposit height, *h*, for each of the *p* = 30 experimental conditions.

The results of the second phase are reported in Figure 9, Figure 10 and Figure 11, which show, for each of the *p* = 30 experimental conditions, the average experimental geometrical parameters (width, depth, and height) and the predicted geometrical parameters estimated by the ANN with nine hidden layer nodes. The Figures indicate that the neural network evaluation accuracy, despite some minor deviations, is generally high, providing for a successful verification of the results obtained in the ANN process parameters estimation. 

## 4. Conclusions

In this research work, laser direct metal deposition of 2024 Al alloy was investigated through an experimental campaign under different process conditions carried out on 5-mm-thick T3 temper 2024 Al alloy plates, generating single deposited metal traces for a length of 100 mm. A critical issue in the laser DMD process is related to the geometrical parameters of the cross-section of the deposited metal trace that should be controlled to meet the part specifications. To this aim, complex mathematical modelling is required including in the model all the relevant processing factors. In this research work, a machine learning approach based on artificial neural networks was developed to find the correlation between the laser DMD process parameters and the output geometrical parameters of the deposited metal trace on 2024 aluminum alloy plates. The specific aim was to identify via ANN the appropriate process parameters required to obtain a deposited trace with given geometrical parameters in terms of width, *w*, depth, *d*, and height, *h*. The results showed that the ANN-based machine learning paradigm is able to accurately estimate the correct laser power, scanning speed and powder feeding rate to achieve a specified geometry for the deposited metal trace, with mean absolute percentage errors of the ANN estimation as low as 2.0% for laser power, 5.8% for scanning speed, and 5.5% for powder feeding rate.

## Figures and Tables

**Table 1:** Main technical features of the laser source.

Parameter	Value
Maximum output power (kW)	4.0
Operating nominal wavelength (nm)	1030
Beam Parameter Product (mm × mrad)	8.0
Core diameter of the delivering fibre (µm)	300
Spot size of the laser beam on the surface (mm)	3.0

**Table 2:** Measured values of the cross-section geometrical parameters for the three repetitions of the diverse experimental process conditions.

Process Conditions			Measured Geometrical Parameters		
*P* (W)	*v* (mm/min)	*ṁ* (g/min)	Width (mm)	Depth (mm)	Height (mm)
1200	400	4	2.93, 3.13, 3.21	0.25, 0.26, 0.25	0.96, 1.02, 0.96
1500	400	4	3.90, 3.78, 3.45	0.48, 0.45, 0.45	1.11, 1.11, 1.02
1500	400	6	3.61, 3.45, 3.78	0.38, 0.36, 0.37	1.43, 1.43, 1.52
1500	400	8	3.69, 3.52, 3.26	0.11, 0.11, 0.11	1.99, 2.06, 1.90
1500	400	10	4.57, 4.30, 4.03	0.20, 0.22, 0.21	2.06, 2.18, 2.18
2000	200	3	5.06, 5.40, 5.26	0.68, 0.68, 0.76	0.84, 0.83, 0.88
2000	200	10	5.30, 5.22, 5.32	0.77, 0.73, 0.74	2.02, 2.12, 2.20
2000	300	3	5.02, 5.48, 5.48	1.87, 1.81, 1.75	1.38, 1.43, 1.33
2000	400	8	4.28, 3.86, 4.07	0.59, 0.60, 0.58	1.78, 1.68, 1.79
2000	500	8	4.01, 4.09, 3.73	0.64, 0.65, 0.66	1.52, 1.44, 1.51
2000	500	3	4.49, 4.12, 4.32	1.21, 1.26, 1.19	1.12, 1.07, 1.15
2500	100	5	11.18, 10.16, 10.77	2.74, 2.91, 2.84	0.85, 0.80, 0.81
2500	150	5	8.95, 8.93, 7.62	1.50, 1.51, 1.52	1.08, 1.06, 0.98
2500	150	7	9.31, 9.32, 8.08	1.51, 1.51, 1.54	2.13, 2.07, 1.99
2500	200	5	7.94, 8.43, 7.78	1.50, 1.53, 1.47	1.07, 1.08, 1.00
2500	200	7	8.25, 8.38, 9.35	1.66, 1.68, 1.52	1.80, 1.82, 2.05
2500	250	5	8.58, 8.54, 7.84	1.71, 1.65, 1.71	0.81, 0.81, 0.73
2500	400	3	5.56, 5.34, 5.00	2.13, 2.11, 2.15	1.12, 1.06, 1.13
3000	150	5	8.95, 9.07, 7.78	2.36, 2.38, 2.53	1.20, 1.11, 1.17
3000	150	7	9.62, 9.49, 9.11	2.59, 2.61, 2.60	1.60, 1.52, 1.47
3000	200	5	8.56, 8.16, 7.44	2.50, 2.43, 2.36	1.00, 1.01, 0.98
3000	200	7	8.66, 8.54, 9.21	2.36, 2.40, 2.47	1.35, 1.28, 1.39
3000	200	4	8.17, 7.82, 8.25	2.39, 2.44, 2.34	0.81, 0.82, 0.74
3000	200	6	9.33, 9.11, 9.23	1.90, 1.85, 1.74	1.16, 1.22, 1.22
3000	200	3	7.24, 7.75, 7.51	2.21, 2.23, 2.37	0.69, 0.66, 0.66
3000	200	10	9.51, 9.91, 8.48	2.39, 2.33, 2.27	1.86, 1.78, 1.95
3000	300	3	6.71, 7.04, 7.37	3.14, 3.31, 3.24	1.05, 1.03, 1.14
3000	500	3	5.23, 5.16, 5.76	2.19, 2.21, 2.35	0.93, 1.00, 0.95
3000	600	3	4.97, 4.68, 4.37	0.94, 0.90, 0.92	0.28, 0.29, 0.30
3000	600	10	5.83, 5.68, 5.00	1.05, 1.06, 1.01	1.30, 1.30, 1.24
